# Pioglitazone abolishes autistic-like behaviors via the IL-6 pathway

**DOI:** 10.1371/journal.pone.0197060

**Published:** 2018-05-23

**Authors:** Thiago Berti Kirsten, Renato C. Casarin, Maria M. Bernardi, Luciano F. Felicio

**Affiliations:** 1 Department of Pathology, School of Veterinary Medicine, University of São Paulo, São Paulo, Brazil; 2 Environmental and Experimental Pathology, Paulista University, São Paulo, Brazil; 3 Graduate Program of Dentistry, Paulista University, São Paulo, Brazil; Max Delbruck Centrum fur Molekulare Medizin Berlin Buch, GERMANY

## Abstract

Autism is characterized by social deficits, communication abnormalities, and repetitive behaviors. The risk factors appear to include genetic and environmental conditions, such as prenatal infections and maternal dietary factors. Previous investigations by our group have demonstrated that prenatal exposure to lipopolysaccharide (LPS), which mimics infections by gram-negative bacteria, induces autistic-like behaviors. No effective treatment yet exists for autism. Therefore, we used our rat model to test a possible treatment for autism. We selected pioglitazone to block or ease the impairments induced by LPS because although this drug was designed as an anti-diabetic drug (it has an insulin effect), it also exerts anti-inflammatory effects. Juvenile offspring were treated daily with pioglitazone, and the main behaviors related to autism, namely, socialization (play behavior) and communication (50-kHz ultrasonic vocalizations), were studied. Biomarkers linked to autism and/or pioglitazone were also studied to attempt to understand the mechanisms involved, namely, IL-6, TNF-alpha, MCP-1, insulin, and leptin. Prenatal LPS exposure induced social deficits and communicational abnormalities in juvenile rat offspring as well as elevated plasma IL-6 levels. Daily postnatal pioglitazone treatment blocked the impairments found in terms of the time spent on social interaction, the number of vocalizations (i.e., autistic-like behaviors) and the elevated plasma IL-6 levels. Thus, pioglitazone appears to be a relevant candidate for the treatment of autism. The present findings may contribute to a better understanding and treatment of autism and associated diseases.

## Introduction

Autism (autism spectrum disorder) is a developmental brain disorder that is characterized by social deficits, communication abnormalities, and cognitive inflexibility and has a higher prevalence in males [1]. One in every 100 children is diagnosed with autism [2, 3]. The risk factors appear to include genetic and perinatal environmental conditions, such as viral prenatal infections and maternal dietary factors; however, the exact etiology remains unknown [4–6].

Previous investigations by our group have demonstrated that the prenatal exposure of rats on gestational day (GD) 9.5 to lipopolysaccharide (LPS; 100 μg/kg, intraperitoneal [i.p.]), which is an endotoxin that mimics infection with gram-negative bacteria, impairs communication and socialization and induces repetitive/restricted behavior in male offspring. However, the behavior of female offspring is not altered [7, 8]. These results suggest that our model of prenatal LPS exposure induces autism-like behavior in offspring [8]. Moreover, we have observed an increase in serum interleukin (IL)-1 beta levels in adult offspring [9], and this finding has previously been reported in several autistic patients [10–12]. Incidentally, the effects of maternal LPS exposure on the developing fetal brain have been suggested to be mediated by the induction of proinflammatory cytokines within the maternal circulation and placenta [13–15].

To date, no effective treatment exists for autism, and there is no consensus regarding the type of medication to prescribe [16]. A few drugs have been approved by the U.S. Food and Drug Administration (FDA), but these agents have limited efficacy, treat only some of the symptoms, and trigger adverse effects [17]. Thus, the purpose of the present study was to use our rat model of autism to test a treatment for autism. We selected pioglitazone as a postnatal treatment to block or ease the impairments induced by prenatal LPS. Pioglitazone is a member of the class of synthetic drugs termed thiazolidinediones, which are agonists of the peroxisome proliferator-activated receptor gamma (PPARγ) [18, 19]. Pioglitazone was originally designed as an anti-diabetic drug due to its insulin sensitizing effect and was approved by the FDA and widely used clinically to treat type 2 diabetes mellitus with few side effects [20]. Thiazolidinediones also exert anti-inflammatory effects in several cell types, and therefore have been considered in the treatment of inflammatory diseases, including atherosclerosis, psoriasis, and inflammatory bowel disease [21–23], and neurological diseases, such as Alzheimer's and multiple sclerosis [24, 25].

Concomitant with other pharmacological and behavioral therapies, pioglitazone induced apparent clinical improvements in autistic patients. In a small cohort of autistic children, daily treatment with pioglitazone eased some autistic behaviors, such as irritability, lethargy, stereotypy, and hyperactivity, without significant side effects [26]. Another pilot double-blind placebo-controlled study indicated positive effects of pioglitazone as an augmentative medication to risperidone in improving the behavioral symptoms of autistic disorder [27]. In the present study, we used our rat model to evaluate whether pioglitazone also exerted beneficial effects, excluding the concomitant therapies used in patients. Moreover, it was important to evaluate other behaviors that are impaired in autism, such as socialization and communication, and to attempt to understand the neuroimmune mechanisms involved. Juvenile offspring were treated daily with pioglitazone, and the main behaviors related to autism, namely, socialization (play behavior) and communication (ultrasonic vocalizations), were studied [1, 28]. Neuroimmune biomarkers linked to autism and/or pioglitazone, namely, IL-6, tumor necrosis factor (TNF)-alpha, monocyte chemotactic protein (MCP)-1/CCL2, insulin, and leptin, were also studied.

## Materials and methods

### Ethics statement

This study was performed in strict accordance with the recommendations in the Guide for the Care and Use of Laboratory Animals of the National Institutes of Health. The protocol was approved by the Committee on the Ethics of Animal Experiments of the School of Veterinary Medicine, University of São Paulo, Brazil (Permit Number: 2824/2012). All efforts were made to minimize the suffering, reduce the number of animals used, and utilize alternatives to in vivo techniques when available. The experiments were performed in accordance with good laboratory practice protocols and quality assurance methods.

### Animals

A total of 19 pregnant Wistar rats between 15 and 17 weeks of age and weighing 220–275 g were used. The rat housing and nutritional conditions, as well as the determination of GD 0 and the handling and care of the dams, were the same as previously described by our group [8]. The dams were allowed to give birth and nurture their offspring under normal conditions. The day of birth was recorded as postnatal day (PND) 1. No handling was performed on PND 1. On PND 2, 6–8 offspring (3–4 males and 3–4 females) were randomly selected for the following studies. The pups remained with each dam until weaning (PND 21). On PND 21, the male rat pups were individually housed in polypropylene cages under the same conditions as their parents. A maximum of two male rats from each litter was used for each offspring/postnatal treatment to minimize the potential confounding factors associated with the litter [29, 30]. Thus, some animals from each LPS litter were assigned to each of the postnatal treatment doses. The female offspring were separated for use in other studies. All of the experiments were performed between 9:30 and 11:00 AM to minimize the effects of circadian rhythms. Testing between groups was intermixed.

### Prenatal treatments

LPS (from Escherichia coli; Sigma-Aldrich, St. Louis, USA; serotype 0127: B8) was dissolved in sterile saline (50 μg/ml LPS in a 0.9% NaCl solution) and administered i.p. to pregnant dams at a dose of 100 μg/kg on GD 9.5. This dose was selected based on our previous findings of maternal sickness behavior and behavioral, brain, and immune impairments in offspring [7, 9, 31]. Other dams received the vehicle (0.9% sterile saline, SAL) on GD 9.5 according to the same treatment schedule as that of the LPS-treated animals. Each control dam was treated with a 0.2 ml/100 g saline solution.

### Postnatal treatments and groups

The offspring that received prenatal LPS or SAL also received pioglitazone or its vehicle dimethyl sulfoxide (DMSO) solution daily from PND 21 until 29. The pioglitazone (Sigma-Aldrich, St Louis, USA) treatment was scheduled in two doses, 0.25 or 1.0 mg/kg/day administered i.p. based on autistic children prescriptions [26], rat studies [32–34], and on the pharmacokinetics of the drug (Actos, Abbott, Rio de Janeiro, Brazil). Daily treatments between PND 21 and 29 were also based on an autistic children study [26] in which more expansive results of pioglitazone were found in younger children (3–5 years old) compared with older children (up to 17 years old). Incidentally, PND 21–29 in rats is approximately the equivalent age of 3–5 years old in humans [35]. DMSO was diluted to 1:10 in SAL.

Four groups were investigated (*n* = 8 per group). (1): the SAL+DMSO group (also referred to as the control group) consisted of offspring that received a prenatal saline injection on GD 9.5 and daily DMSO solution injections between PND 21 and 29; (2) the LPS+DMSO group (also referred to as the LPS group) consisted of offspring that received a prenatal LPS injection on GD 9.5 and daily DMSO solution injections between PND 21 and 29; (3) the LPS+PI0.25 group consisted of offspring that received a prenatal LPS injection on GD 9.5 and pioglitazone (0.25 mg/kg/day injections) between PND 21 and 29; and (4) the LPS+PI1.0 group consisted of offspring that received a prenatal LPS injection on GD 9.5 and pioglitazone (1.0 mg/kg/day) between PND 21 and 29.

### Play behavior

Impaired social interaction and play behavior are some of the most typical symptoms of autism [1]. These behaviors were evaluated using the play behavior test that was based on our previous studies [7, 8]. Briefly, on PND 21, the rat pups were individually housed in polypropylene cages under the same conditions as their parents until PND 30. The rationale behind the social isolation was to increase the motivation to initiate play behavior [36]. Play behavior was evaluated on PND 30 because this behavior has been demonstrated to peak during this time [37]. For the evaluation, each isolated rat in the four groups was paired with a naïve male rat (i.e., without any treatment) that was previously housed in a group environment. The weight difference of the two rats (isolated and naïve-grouped) was up to 10 g. Each naïve rat was only used for one pairing. The testing room was small and dimly lit with a controlled temperature of 22°C ± 2°C and with a video camera mounted near the ceiling to record the behavior. A 5-min period was allowed for the animals to adapt to the testing room prior to matching. The naïve-grouped rat was always placed in the cage of the isolated rat in which the test was conducted; therefore, the isolates are also referred to as the residents, and the naïve-grouped rats are referred to as the intruders. Their behaviors were recorded for 10 min in the testing room isolated from the experimenter. The behavioral analyses were conducted using the videotaped recordings by a single observatory who was blind to the treatments. To distinguish the residents and intruders during the behavioral analyses, the tails were painted in different colors and patterns with non-toxic pens during the body weight measurement. The following parameters were measured over each 10-min session only for the isolated rats: pinning frequency (the total number of times the resident rat laid on its back and showed his belly to the intruder, which mounted the resident from above to complete the social interaction), darting frequency (the total number of times the resident moved rapidly towards, in parallel, or away from the intruder), rearing frequency (the total number of times the resident rat stood on its hind legs without interacting with the intruder), and total time (in s) spent with social interactions (which included the time spent pinning, sniffing, following, and crawling over/under the partner). Pinning was considered social play, darting was considered play solicitation, and total social interaction included social investigations, solicitation, and play, and rearing was considered a non-social exploratory behavior [37].

### Ultrasonic vocalization

Impaired communication is one of the most typical symptoms of autism [1]. Juvenile rats emit 50 kHz ultrasonic vocalizations in response of social appetitive stimuli, such as play behavior [38, 39]. Fifty kilohertz ultrasonic vocalization evaluation may also be related to autistic rat models [28, 40], but this relation lacks further studies. Thus, we assessed the 50-kHz ultrasonic vocalizations of the juvenile rats immediately after the 10-min play behavior test (PND 30). Evaluating ultrasonic vocalizations during play behavior test would prevent distinguishing changes between each rat during pairing, i.e., if any impairment appeared from some specific experimental group and not from the naïve rats. The rats were individually placed in the center of an open field device that included a round wooden arena (40 cm in diameter, 25.5 cm high walls) painted with an acrylic washable covering. The following parameters were automatically measured using an Ultravox system (Noldus Information Technology, Wageningen, Netherlands, composed of software, a filter, and an ultrasonic microphone that was tuned to a range centered at 50 kHz and placed next to the inner wall of the arena, 8 cm away from its floor) over a period of 5 min: the number of vocalizations, the total time spent vocalizing, the mean vocalization duration, the maximal vocalization duration, the total silence duration, the mean silence duration interval, and the maximal silence duration interval. The durations were recorded in seconds. The testing room was small, dimly lit and had a controlled temperature of 22°C ± 2°C with specific foam installed in the walls for acoustic insulation to isolate the experimenter and the other rats. The open field device was washed with a 5% alcohol/water solution before the placement of the animals to obviate the possible biasing effects from odor clues left by the earlier rats.

### Plasma evaluations

On PND 36–40, the rats that were previously evaluated for their behavior were decapitated, and their trunk blood was collected in conical tubes that contained 10% ethylenediaminetetraacetic acid (EDTA). The samples were centrifuged (3,500 RPM, 15 min, 15°C), and the plasma was obtained. Using the Luminex/Magpix system (RSH69K03, Millipore, Billerica, USA), biomarkers related to autism and/or pioglitazone were studied, including the following: IL-6, TNF-alpha, MCP-1, insulin, and leptin (part of the Milliplex map rat metabolic hormone magnetic bead panel-metabolism multiplex assay, catalogue no. RMHMAG-84K; Millipore, Billerica, USA) according to the manufacturer's instructions. The range of detection of the peptides was 1.6–400.000 pg/ml. The concentrations were estimated using a five-parameter polynomial curve (Xponent software, Millipore). All results are expressed in pg/ml.

### Statistical analysis

Homogeneity and normality were verified using a Bartlett’s test. One-way analysis of variance (ANOVA) followed by Newman-Keuls multiple comparison tests were used to compare the parametric data between the four groups. A Spearman's rank-order correlation was performed for the correlational analyses between the behavioral and peripheral factors (correlations within each treatment group and across the groups). The results are expressed as the mean ± the SEM. In all cases, the results were considered as statistically significant at *p* < 0.05.

## Results

**Fig 1:**
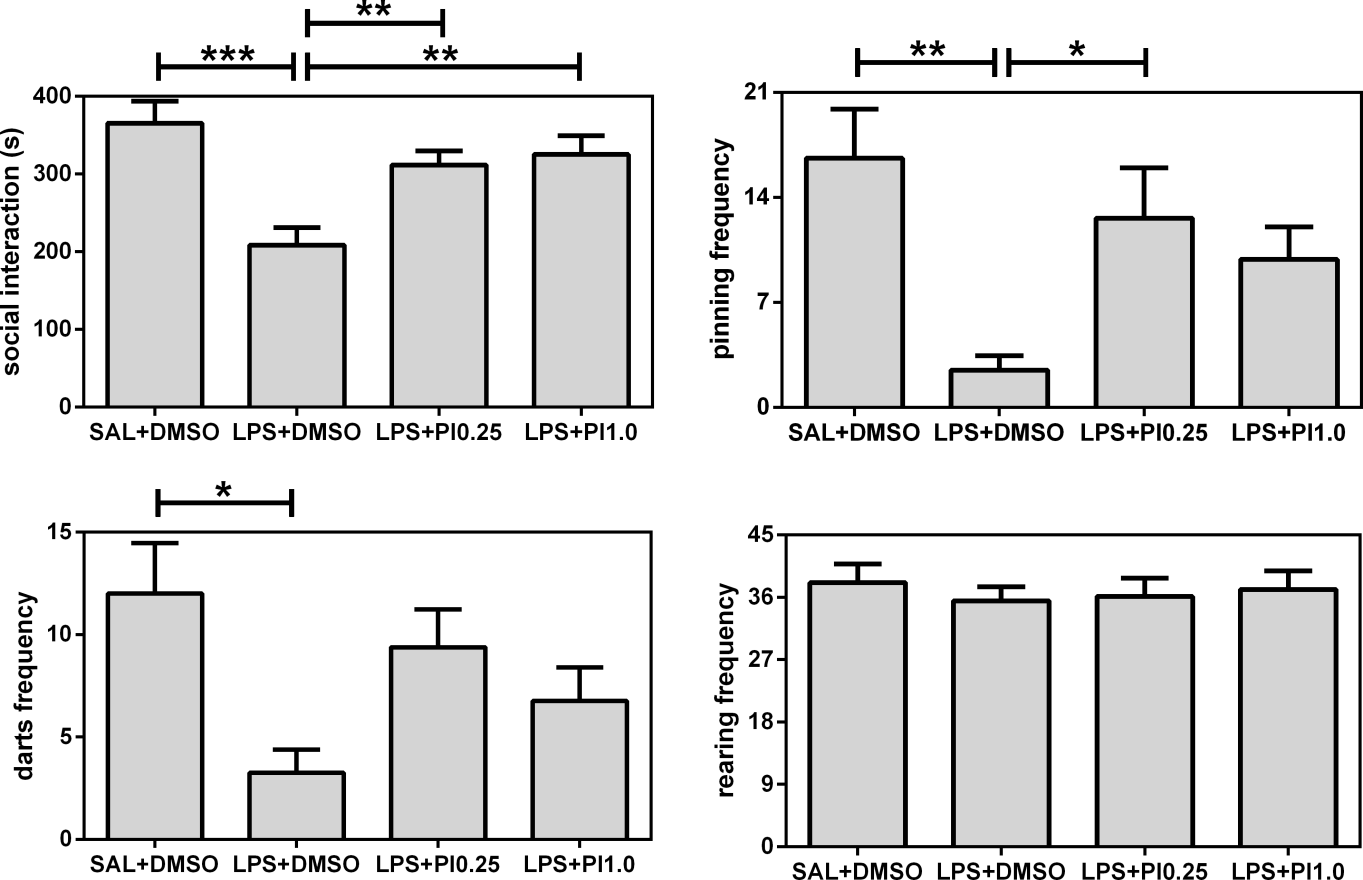
Play behaviors. The effects of prenatal LPS (100 μg/kg at gestational day 9.5) and postnatal pioglitazone (0.25 and 1.0 mg/kg/day between postnatal days 21 and 29) exposures on play behaviors in juvenile male rat offspring. SAL+DMSO, prenatal saline injection and postnatal daily DMSO injection; LPS+DMSO, prenatal LPS injection and postnatal daily DMSO injection; LPS+PI0.25, prenatal LPS injection and postnatal pioglitazone 0.25 mg/kg/day; LPS+PI1.0, prenatal LPS injection and postnatal pioglitazone 1.0 mg/kg/day (*n* = 8 rats/group). *p < 0.05, **p < 0.01, and ***p < 0.0001 (one-way ANOVA followed by the Newman-Keuls test). The data are expressed as the mean ± the SEM.

Fig 1 shows the effects of prenatal LPS and postnatal pioglitazone exposure on the play behavior of the rats. All of the social parameters were affected by the treatments: social interaction (F(3/28) = 8.07, *p* = 0.0005), pinning (F(3/28) = 5.15, *p* = 0.0058), and darts (F(3/28) = 4.12, *p* = 0.0153). However, the non-social exploratory activity, namely, rearing (F(3/28) = 0.21, p = 0.8910), was not affected. Specifically, prenatal LPS exposure (LPS+DMSO group) impaired play behavior in the rats; it reduced social interaction and pinning as well as darts compared with the control group (SAL+DMSO). Post-treatment with pioglitazone increased play behavior in the rats that were prenatally exposed to LPS; both doses of pioglitazone (groups LPS+PI0.25 and LPS+PI1.0) exhibited increased social interaction compared with the LPS+DMSO group and reached the same levels exhibited by the control group. Regarding pinning, only the lowest dose of pioglitazone (group LPS+PI0.25) was able to increase pinning compared with the LPS+DMSO group and reach the same levels exhibited by the control group. Darting frequency was not recovered after the pioglitazone treatments. Thus, prenatal LPS impaired social play, play solicitations, and the social investigations of the rats, and pioglitazone treatment blocked the social interactions and pinning impairments. These effects were specific for the social and not the motor/exploratory performance.

**Fig 2:**
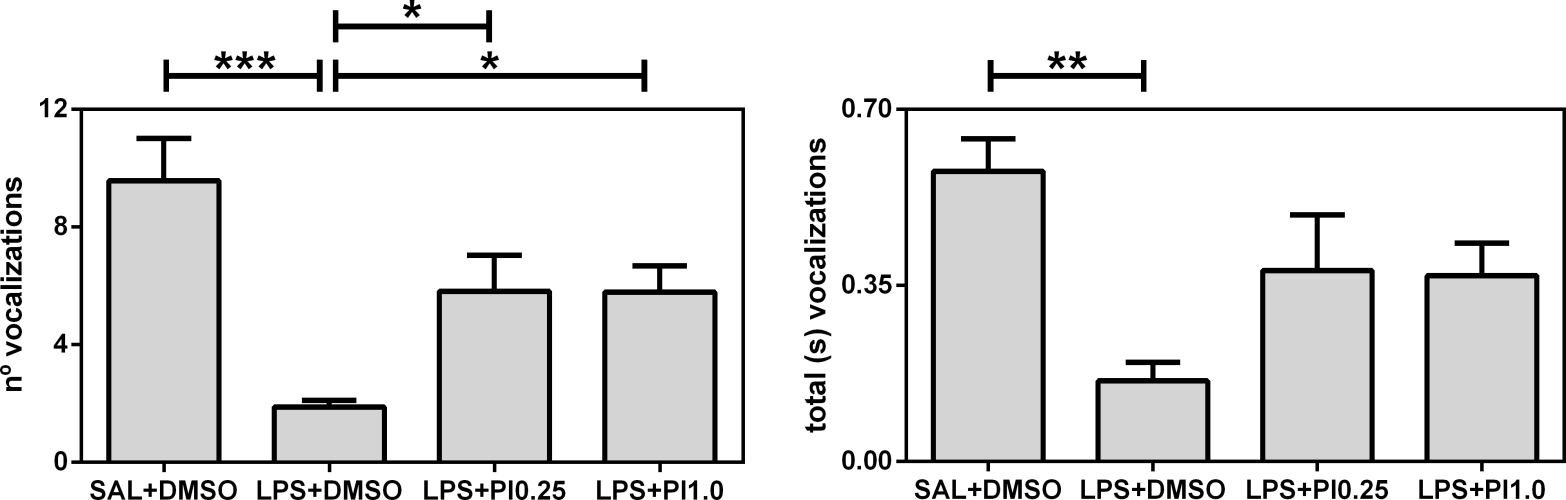
Ultrasonic vocalizations. The effects of prenatal LPS (100 μg/kg at gestational day 9.5) and postnatal pioglitazone (0.25 and 1.0 mg/kg/day between postnatal days 21 and 29) exposures on 50-kHz ultrasonic vocalizations in juvenile male rat offspring. SAL+DMSO, prenatal saline injection and postnatal daily DMSO injection; LPS+DMSO, prenatal LPS injection and postnatal daily DMSO injection; LPS+PI0.25, prenatal LPS injection and postnatal pioglitazone 0.25 mg/kg/day; LPS+PI1.0, prenatal LPS injection and postnatal pioglitazone 1.0 mg/kg/day (*n* = 8 rats/group). *p < 0.05, **p < 0.01, and ***p < 0.0001 (one-way ANOVA followed by the Newman-Keuls test). The data are expressed as the mean ± the SEM.

Fig 2 shows the effects of prenatal LPS and postnatal pioglitazone exposures on the 50 kHz ultrasonic vocalizations of the rats. The number of vocalizations (F(3/28) = 9.00, *p* = 0.0002) and the total time spent vocalizing (F(3/28) = 5.25, *p* = 0.0053) were affected by the treatments. Specifically, prenatal LPS exposure (LPS+DMSO group) reduced both the number and total time of vocalizations compared with the control group (SAL+DMSO). Post-treatment with pioglitazone increased the number of ultrasonic vocalizations in the rats that were prenatally exposed to LPS; both doses of pioglitazone (groups LPS+PI0.25 and LPS+PI1.0) increased the number of vocalizations compared with the LPS+DMSO group. However, the pioglitazone treatments were not able to reach the levels exhibited by the control group in terms of the number of vocalizations and were not able to correct the LPS effects on the total time of vocalizations. The other parameters (mean and maximal vocalization duration, and total, mean, and maximal silence duration) were found to be similar among the four groups (data not shown). Thus, prenatal LPS impaired the numbers of 50-kHz ultrasonic vocalizations of rats, and pioglitazone treatment partially blocked these impairments.

**Fig 3:**
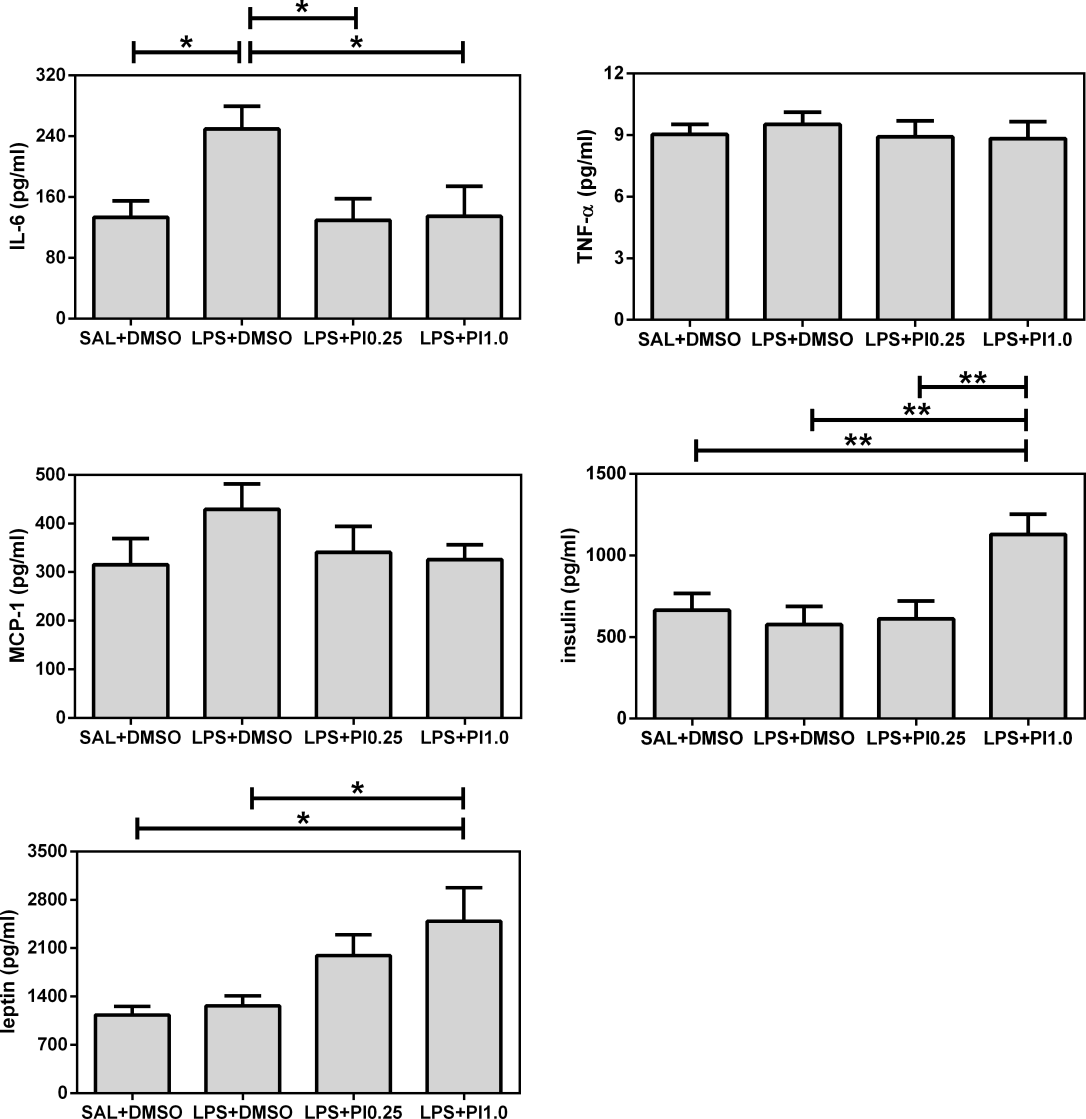
Plasma evaluations. The effects of prenatal LPS (100 μg/kg at gestational day 9.5) and postnatal pioglitazone (0.25 and 1.0 mg/kg/day between postnatal days 21 and 29) exposures on IL-6, TNF-alpha, MCP-1, insulin, and leptin plasma levels in juvenile male rat offspring. SAL+DMSO, prenatal saline injection and postnatal daily DMSO injection; LPS+DMSO, prenatal LPS injection and postnatal daily DMSO injection; LPS+PI0.25, prenatal LPS injection and postnatal pioglitazone 0.25 mg/kg/day; LPS+PI1.0, prenatal LPS injection and postnatal pioglitazone 1.0 mg/kg/day (*n* = 8 rats/group). *p < 0.05 and **p < 0.01 (one-way ANOVA followed by the Newman-Keuls test). The data are expressed as the mean ± the SEM.

Fig 3 shows the effects of prenatal LPS and postnatal pioglitazone exposure on the IL-6, TNF-alpha, MCP-1, insulin, and leptin plasma levels of the rats. The IL-6 levels were affected by the treatments (F(3/28) = 3.68, *p* = 0.0237). Prenatal LPS exposure (LPS+DMSO group) increased the IL-6 levels compared with the control group (SAL+DMSO). Both doses of the post-treatment with pioglitazone decreased the IL-6 levels in the rats that were prenatally exposed to LPS (groups LPS+PI0.25 and LPS+PI1.0) compared with the LPS+DMSO group, which reached the same levels exhibited by the control group. Neither the TNF-alpha nor MCP-1 levels were affected by the treatments (F(3/28) = 0.20, *p* = 0.8941 and F(3/28) = 1.15, *p* = 0.3449, respectively). However, both the insulin and leptin levels were affected by the treatments (F(3/28) = 5.31, *p* = 0.0050 and F(3/28) = 4.45, *p* = 0.0112, respectively). Specifically, only the pioglitazone treatment of 1.0 mg/kg/day associated with LPS (group LPS+PI1.0) affected the insulin and leptin levels by increasing them compared with the other groups.

Based on the results found in the one-way ANOVA test, we selected the most relevant behavioral and peripheral factors for the correlational analyses, namely, the social interaction, number of vocalizations, and IL-6 levels. Preliminary analyses revealed that there were no monotonic relationships as assessed by visual inspection of a scatterplot in the analyses between the IL-6 levels and the total time spent on social interactions. Similarly, there were no monotonic relationships in the analyses between the IL-6 levels and the numbers of vocalizations. Thus, the correlational analyses were not performed.

## Discussion

Prenatal infection/inflammation on GD 9.5 falls within a critical period for brain organogenesis. Infections associated with immunological events during the early/middle fetal stages (e.g., GD 8–10 in rats and mice) may have a stronger influence on neurodevelopment than infections that occur during late-stage pregnancy. Maternal immune activation during early/middle pregnancy may interfere with cell proliferation, differentiation, migration, target selection, and synapse maturation, which may later lead to multiple brain and behavioral abnormalities in adulthood [41–44]. Previous data from our group have corroborated that GD 9.5 is a critical period. We have demonstrated that prenatal exposure to LPS on GD 9.5 in rats induces short- and long-term reproductive, behavioral, and neuroimmune impairments in the offspring [7–9, 31, 45–47].

The behavioral impairments induced in our rat model have been particularly associated with autistic-like behaviors [7, 8, 30, 48]. Our present findings of prenatal LPS exposure inducing social deficits evaluated in the play behavior test and communication abnormalities in the 50-kHz ultrasonic vocalization test corroborate this hypothesis. Specifically, we demonstrated impaired social play, play solicitations, and social investigations, as well as fewer vocalization responses to social appetitive stimuli of juvenile rats. Therefore, our model of prenatal LPS exposure induced variations in the main behavior deficits of autism that have been experimentally studied in rodent models [28, 37, 40] and found in patients [1].

Incidentally, epidemiological studies and experimental animal models have indicated an association between maternal immune activation/infection during pregnancy and an increased risk of central nervous system disorders in the offspring, including schizophrenia, autism, and cerebral palsy [49–52]. For example, a well-established animal model is based on prenatal treatment with the viral-mimic inflammatory agent polyriboinosinic-polyribocytidilic acid (poly[I:C]), which is a synthetic analog of double-stranded RNA. Prenatal poly (I:C) exposure in mice is a powerful experimental tool to induce and investigate the distinct brain and behavioral abnormalities associated with schizophrenia and with early/middle (GD 9) and late (GD 17) exposure being relevant to the positive and negative cognitive symptoms, respectively [42, 53–55].

Prenatal/perinatal exposures to numerous pathogens, including rubella, measles, and cytomegalovirus, have been implicated in the etiology of autism, which suggests that the infection-associated risk of autism might not be pathogen specific [49]. This hypothesis is supported by a hospital study that suggested that the maternal exposure to various viral or bacterial infections significantly increases the risk of autism-spectrum disorders in children, and this effect appears to be unrelated to hospitalization per se [50]. Thus, acute fetal neuroinflammation, together with its effects on early neurodevelopmental processes, may facilitate the development of the psychopathological and neuropathological phenotypes of autism [49].

Other evidence of the intrinsic relation between neuroinflammation and autism include elevated levels of blood and brain proinflammatory cytokines in autistic patients. Although different cytokines, such as IL-1 and TNF-alpha, are elevated in the samples [10, 12], IL-6 seems to play a key role in the mechanistic pathway [56–58]. We also found elevated IL-6 levels in our rat model after prenatal LPS exposure.

We selected pioglitazone as a postnatal treatment to block or ease the impairments induced by prenatal LPS because of the promising results in the treatment of some symptoms of autistic children [26]. We did not consider prenatal treatment with pioglitazone to prevent/ameliorate the effects of prenatal LPS. It is known that the gestational environment is very sensitive to immunological changes, as its use during pregnancy is not recommended according to manufacturer's information (Actos, Abbott, Rio de Janeiro, Brazil).

Postnatal treatment with pioglitazone partially blocked the behavioral and immune impairments induced by prenatal LPS exposure. Both doses of pioglitazone (0.25 and 1.0 mg/kg/day, between PND 21 and 29) ameliorated the social interaction and vocalization deficits and the increase in the IL-6 levels induced in our rat model of autism. Thus, pioglitazone treatment blocked some of the autistic-like effects in rats. Taken together, the daily administration of pioglitazone may be suggested for the treatment of autism not only for reversing the irritability, lethargy, stereotypy, and hyperactivity [26] but also for socialization and communication impairments. Because Boris et al [26] used other pharmacological and educational therapies together with pioglitazone, we think that these are the first results of beneficial aspects of pioglitazone in autism without other important interferents/variables.

In vitro studies using astrocytes obtained from the cerebral cortices of newborn C57BL/6 mice and grown in culture that were stimulated with LPS have demonstrated that pioglitazone treatment inhibits the secretion of proinflammatory factors, such as nitric oxide and IL-6, and enhances the levels of the secretion of anti-inflammatory factors IL-4 and IL-10 [59]. Therefore, considering the results of Qiu and Li [59] and our present findings, pioglitazone acted to benefit autistic-like behaviors possibly via the inhibition of IL-6 secretion in astrocytes stimulated by LPS, which inhibited the neuroinflammatory response.

The insulin analyses were performed because of their relation with both pioglitazone and autism. Classically, pioglitazone has an insulin-sensitizing effect and is widely used clinically to treat type 2 diabetes mellitus [20, 60]. Indeed, 1.0 mg/kg/day of pioglitazone increases insulin levels. Although there are some studies that have attempted to relate diabetes/insulin to autism, the evidence is limited [61, 62]. In the study of Boris et al [26], over the course of pioglitazone treatment, there were no elevations of the insulin levels of autistic children. We also did not find changes in the insulin levels in the autistic-like rats. Thus, the induction of the autistic-like effects after prenatal LPS exposure and the beneficial effect of the pioglitazone treatment appear not to be related to the insulin pathway.

The leptin analyses were performed because of their relation with both autism and pioglitazone. Some autistic patients present elevated levels of plasmatic leptin [63, 64]. However, we did not find any significant differences in plasma leptin levels after prenatal LPS exposure. In contrast, 1.0 mg/kg/day of pioglitazone increased then leptin levels. The literature demonstrates contradictory findings regarding leptin levels after pioglitazone treatment. For example, three [65] or four [66] months of pioglitazone treatment are not able to change leptin levels in patients with type 2 diabetes mellitus. In another study, while then baseline concentrations of leptin were not different, after three months, pioglitazone decreased the leptin concentrations in men with type 2 diabetes mellitus. Moreover, patients with metabolic syndrome and multiple sclerosis present with decreased leptin levels after pioglitazone treatment [67]. Thus, the induction of the autistic-like effects after the prenatal LPS exposure and the beneficial effect of the pioglitazone treatment appear to not be related to the leptin pathway.

This study has some limitations. First, other behavioral aspects found in the autistic-like rats should be studied, specifically the repetitive behaviors. Second, we focused our present study on the peripheral mediators involved in prenatal LPS exposure and the rat model of autism. Future studies focusing on the central nervous system (e.g., neuroinflammatory pathways and the production of IL-6 by astrocytes) would be interesting to improve the understanding of the possible mechanisms involved with autism. Moreover, experiments examining whether pioglitazone directly acts through IL-6 should be performed in future studies. Finally, the inclusion of a pioglitazone control group (saline+pioglitazone) would demonstrate any effects of the drug alone and strengthen the findings of the manuscript.

In conclusion, prenatal LPS exposure on GD 9.5 induced social deficits and communicational abnormalities in juvenile rat offspring, namely, autistic-like behaviors. We also found elevated plasma IL-6 levels in our rat model, which has been considered to be one of the key mechanistic pathways in autism [56–58]. Daily postnatal pioglitazone treatment blocked the impairments found in terms of the time spent on social interaction, the number of vocalizations (i.e., autistic-like behaviors) and the elevated plasma IL-6 levels induced by LPS exposure in the offspring. Thus, pioglitazone appears to be a relevant candidate for the treatment of autism. There are no effective treatments for autism to date [16], and the currently available drugs have limited efficacies and trigger adverse effects [17]. The present findings may contribute to a better understanding and treatment of autism and associated diseases.
